# Can we predict functional decline in hospitalized older people admitted through the emergency department? Reanalysis of a predictive tool ten years after its conception

**DOI:** 10.1186/s12877-017-0498-0

**Published:** 2017-05-12

**Authors:** Isabelle De Brauwer, Pascale Cornette, Benoît Boland, Franck Verschuren, William D’Hoore

**Affiliations:** 1 Institute of Health and Society (IRSS), Université catholique de Louvain, Brussels, Belgium; 2 Department of Geriatric Medicine, Cliniques universitaires Saint Luc, Brussels, Belgium; 3 Department of Acute Medicine, Cliniques universitaires Saint Luc, Brussels, Belgium

**Keywords:** Older people, Emergency department, Screening tool, Functional decline

## Abstract

**Background:**

In the Emergency Department (ED), early and rapid identification of older people at risk of adverse outcomes, who could best benefit from complex geriatric intervention, would avoid wasting time, especially in terms of prevention of adverse outcomes, and ensure optimal orientation of vulnerable patients. We wanted to test the predictive ability of a screening tool assessing risk of functional decline (FD), named SHERPA, 10 years after its conception, and to assess the added value of other clinical or biological factors associated with FD.

**Methods:**

A prospective cohort study of older patients (*n* = 305, ≥ 75 years) admitted through the emergency department, for at least 48 h in non-geriatric wards (mean age 82.5 ± 4.9, 55% women). SHERPA variables (i.e. age, pre-admission instrumental Activity of Daily Living (ADL) status, falls within a year, self-rated health and 21-point MMSE) were collected within 48 h of admission, along with socio-demographic, medical and biological data. Functional status was followed at 3 months by phone. FD was defined as a decrease at 3 months of at least one point in the pre-admission basic ADL score. Predictive ability of SHERPA was assessed using c-statistic, predictive values and likelihood ratios. Measures of discrimination improvement were Net Reclassification Improvement and Integrated Discrimination Improvement.

**Results:**

One hundred and five patients (34%) developed 3-month FD. Predictive ability of SHERPA decreased dramatically over 10 years (c = 0.73 vs. 0.64). Only two of its constitutive variables, i.e. falls and instrumental ADL, were significant in logistic regression analysis for functional decline, while 21-point MMSE was kept in the model for clinical relevance. Demographic, comorbidity or laboratory data available upon admission did not improve the SHERPA predictive yield.

**Conclusions:**

Prediction of FD with SHERPA is difficult, but predictive factors, i.e. falls, pre-existing functional limitation and cognitive impairment, stay consistent across time and with literature. As accuracy of SHERPA and others existing screening tools for FD is moderate, using these predictors as flags instead of using composite scales can be a way to screen for high-risk patients.

**Electronic supplementary material:**

The online version of this article (doi:10.1186/s12877-017-0498-0) contains supplementary material, which is available to authorized users.

## Background

Older people represent an increased proportion - currently 12–24% - of all emergency department (ED) admissions [1–3]. This specific population is at increased risk of adverse events, e.g. functional decline, institutionalization and death [1–3]. In particular, one third to one half of them require admission to an acute setting [1, 2]. Ellis showed that Comprehensive Geriatric Assessment (CGA) performed in dedicated settings, i.e. Geriatric Evaluation and Management Unit (GEMU), improves outcomes for the frail older patients [4]. Early screening for frailty would therefore allow avoiding wasting time, especially in terms of prevention of adverse outcomes, and ensure optimal orientation of frail patients. Given the lack of validated definition of frailty at hospital admission, the risk of functional decline (FD) should be early assessed [5, 6]. FD is indeed a frailty-related adverse outcome and has important public health implications, i.e. in terms of services use [4].

FD screening tools for older inpatients were recently reviewed [7–9]. Predictive accuracy of these tools was moderate and few of these tools were validated in different settings. Moreover many of them were developed in the late nineties. Health policy, clinical practice and target population - 65+ vs. 75+ − changed over this period of time, which may affect the predictive performance of these screening tests [10–12].

In order to optimally allocate dedicated interventions to frail older people, aged 75 years and over, admitted through ED, we wanted to test the accuracy of predicting FD of one of them - the SHERPA screening tool [13] - ten years after its conception. According to de Saint Hubert, SHERPA has practical interest and, among other tools, the larger area under curve of Receiver Operating Characteristics (ROC) curve, a common measure of predictive accuracy [7–9]. This is the first in-depth reanalysis of the accuracy of SHERPA in predicting functional decline. In this sense, this is also the first validation of SHERPA on a separate data set, taking into account the fact that the profile of hospitalized elderly may have changed. In a second step, as suggested by some authors, we wanted to assess the added predictive value of different factors, e.g. biological parameters or comorbidity, which are associated to FD [7–9].

## Methods

### Study design and patient population

We conducted a prospective observational cohort study in a 900-bed Belgian University hospital, in an urban area.

Patients aged seventy-five years and older admitted for at least forty-eight hours in a medical or a surgical ward through the Emergency Department (ED) were eligible. Exclusion criteria were admission to GEMU, short life expectancy (< 3 months), complete functional dependence for all the six Activities of Daily Living (ADL) based on retrospective report [14], admission to an intensive care unit or admission for a major stroke. The two last exclusion criteria are particular conditions associated with a very high risk of FD, while the first one is known to prevent FD [4]. Informed consent was obtained from the patient or the caregiver in case of cognitive impairment. The study protocol was approved by the Ethics Committee of our hospital (BE4032008488).

### SHERPA

SHERPA (Score Hospitalier d’Evaluation du risque de Perte d’Autonomie) [13] assesses the risk of FD following an unscheduled hospitalisation through the ED, regardless of the presenting complaints or ED diagnosis. It uses five patient characteristics, easy to access by the mean of admission interview, i.e. age, fall (s) within the last year, performance in instrumental activities of daily living (iADL) [15], an abbreviated Mini Mental State (21-point MMSE) assessing cognitive ability [16], and self-perceived health. SHERPA (score range: 0–11.5) classifies older ED patients into 4 risk categories of risk of FD: low, moderate, intermediate, or high.

SHERPA is closely related to HARP, a screening tool that was also tested for discrimination in this study [17]. In this observational study, no other specific screening tool and no specific intervention other than usual care were used for the management of older people in the ED or in any other hospital care unit where the eligible patients were admitted. SHERPA scores were only calculated at the end of the study.

### Data collection and measures

From December 2008 to December 2009, one medical doctor (IDB) trained in geriatric medicine performed the interview within forty-eight hours after ED admission, according to a Comprehensive Geriatric Assessment (CGA) protocol. Caregiver was interviewed if the patient was cognitively impaired. Upon admission, patients were assessed for pre-hospital (two weeks before admission) functional independence using the six dichotomized basic ADL domains (bADL, six-point Katz scale, i.e., bathing, dressing, walking, toileting, continence and eating) [14] and the iADL ones (seven-point Lawton scale, i.e. telephoning, shopping, preparing meals, doing housework, using transportation, managing finances and taking medications) [15]; the higher the bADL or iADL score, the higher the independence. Socio-demographic and medical data were also collected. Comorbidity was assessed from patient computerized record and calculated according to the Cumulative Illness Rating Scale for Geriatrics (CIRS-G score) [18]. Number of medications before admission was also recorded. Albumin (g/dL), estimated GFR (Modification of diet in renal disease, MDRD, mL/min), C-reactive protein (CRP, mg/dL) and haemoglobin (g/dL) were collected from admission blood sample (within 48 h). The main discharge diagnosis of the hospital stay was coded following the chapters of International Classification of Disease, Ninth Revision, Clinical Modification (ICD-9-CM). The same researcher followed up by phone all patients at 3 months for place of living*,* rehospitalisation, rehabilitation stay, ADL status, and death. Characteristics of 1999 cohort -enrolled for the development of SHERPA have been already described [13]. However, for the sake of comparison, only patients aged 75 years and over of the 1999 cohort were included in this study.

### Outcome variable: functional decline at 3 months

Functional decline was the main outcome, and was defined as a decrease of at least one point in ADL score (range 0–6) between preadmission and 3-month post-discharge status. To avoid bias, the SHERPA score was calculated after the end of the follow-up.

### Statistical Methods

#### Reliability

Intra- and inter-reliability of SHERPA was analysed in an independent sample of thirty older patients, enrolled prospectively. The only exclusion criterion was the impossibility of interview because of clinical or cognitive status. Researchers (IDB, PC) were blinded to previous SHERPA evaluation of the patient and SHERPA scores were calculated after the end of the protocol. We used IBM SPSS Statistics software, version 19. Intraclass correlation coefficients (ICC) and their 95% confidence interval (IC95%) were calculated using a two-way random effects model [19].

#### Sample size

In the study by Cornette et al. [13], the rate of the proportions of decliners between the two high-risk classes and the two low-risk classes was 3.06 [13], (Table 3). We expected that this rate was presently 2 at most. Based on this estimate, with a 5% alpha level and a 80% power, the total sample size was 232 [20]. We decided to include about 300 patients, a conservative number taking into account missing data, deaths and other lost at follow-up, and lack of informed consent.

Analysis were performed on the complete data as regard SHERPA (missed data, *n* = 3)

#### Model fit and discrimination

Descriptive statistics were performed for social, demographic, functional and clinical characteristics of the patients. Means (standard errors of the mean) and percentages were used for continuous and categorical variables respectively.

The development of the original SHERPA score [13] was based on the following strategy. Firstly, an extensive literature review, complemented by a DELPHI study among Belgian French-speaking geriatricians, was performed to identify candidate prognostic variables. This step resulted in the selection of 50 prognostic variables. Secondly, data were collected on 625 patients admitted to 2 Belgian hospitals via the emergency department, of which 552 patients could be followed up at 3 months from discharge. A subset of 480 patients was available with complete data on functional items (especially the mini-mental state score, which was missing for 70 patients). Thirdly, univariate analyses were performed and resulted in the selection of 12 variables which were statistically significantly associated with functional decline at 3 months post-discharge (Table 2 *in* [13]). Fourthly, a parsimonious logistic model including 5 relevant and statistically significant variables was identified. This modelling was “part science, part statistical method, and part experience and common sense” [21]. Practically, we used iteratively backward selection to exclude clinically and statistically unimportant variables, checking changes in regression coefficients and changes in likelihood ratio tests. Fifth, for the sake of making a clinical use easier, the SHERPA scoring was developed according to the final regression model of step 4 [13].

In the present paper, we collected data on a new cohort of patients admitted to one of the 2 hospitals surveyed by Cornette. Steps 1 and 2 of Cornette study were not replicated due to the fact that this study was not aimed at developing a new score. Data analysis was identical to the Cornette study.

We used multivariate logistic regression to assess the calibration and discrimination of the synthetic SHERPA score (four-level score) and its constitutive variables, i.e. age (75–79, 80–84 and >85 years old), falls in previous year (yes-no), self-rated health (good-bad), iADL (0–2, 3–4, 5, 6–7) and 21-point MMSE (> or = 15 vs. < 15), with FD as the dependent variable. Firstly, the logistic regression model was used to calibrate the SHERPA score on the 2009 cohort and to compare calibration and discrimination with values found on the 1999 cohort (development cohort). Calibration was assessed with the Hosmer-Lemeshow statistic and discrimination was assessed with the c-statistic [22]. Sensitivity, specificity, predictive value and likelihood ratio were calculated at all cut-off of the SHERPA 4-level scale.

Secondly, candidate variables were tested to possibly improve model calibration and discrimination. Candidate variables information had to be available at or near admission to the emergency department and these variables were selected as recommended by the literature [6, 7, 9]. We also tested stay in intensive care unit (ICU; admission during the course of hospital stay) and discharge diagnosis to assess the association of disease with 3-month FD. Due to the availability of these data, the analyses were only possible for the 2009 cohort.

Goodness-of-fit was assessed with the Bayes Information Criterion (BIC) while measures of discrimination improvement were Net Reclassification Improvement (NRI) and Integrated Discrimination Improvement (IDI) [22–24]. The SAS macro provided by Cook was used to compute these indices [23]. All analyses were performed with SAS (9.2 release).

## Results

### Population characteristics and functional decline

**Fig. 1:**
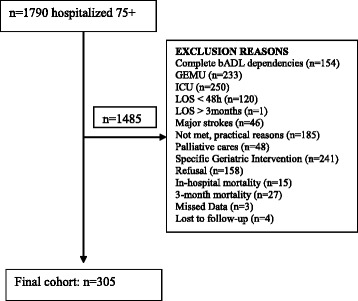
Inclusion Tree bADL: basic Activity of Daily Living; GEMU: Geriatric Evaluation and Management Unit; ICU: Intensive Care Unit; LOS: Length Of Stay

Figure 1 summarized the inclusion process from which finally, data from 305 patients were analysed. Participant features are summarized in Additional file 1: Table S1.

Patients (mean age 82 years, 55% women) were mainly hospitalized in internal medicine, cardiovascular (not surgical) or orthopaedic wards (25, 19 and 17%, respectively). The three main discharge diagnoses (ICD-9-CM chapters) belonged to the circulatory system (31%), the digestive system (14%), or to “injury and poisoning”, including falls (24%). For the latter diagnosis group, 49 patients (66%) were hospitalized in an orthopaedic ward, in which 98% (*n* = 48) had suffered from a fall within the previous year. All but two of the 49 patients admitted in orthopaedic wards presented a fracture, of which 74% were hip fractures.

After 3 months, FD had occurred in 33% (*n* = 102) patients, according to the above definition. Within this period, nursing home admission and hospital return rates were respectively 8% (*n* = 24) and 27% (*n* = 83).

### Intra- and inter-reliability

ICC were respectively 0.961 (95% Confidence Interval (CI) [0.921–.981]) and 0.995 (95%CI [0.990–0.998]) for intra and inter-reliability.

### Model fit and discrimination

Discrimination ability of logistic model was lower in the 2009 cohort than in the 1999 cohort (c-statistic = 0.64 vs. 0.73, *p* > 0.10). The same was observed for the SHERPA 4-level scale; the distribution of risk of FD among the SHERPA categories was much more uniform in 2009 cohort than in 1999 cohort (Table 1). Table 2 summarizes the performance of SHERPA 4-level scale at its three cut-off points, and shows a poor predictive ability. Only the cut-off >3 showed a good specificity (88%) and a positive likelihood ratio upper 2, but with an 18% prevalence of positive test and 72% of decliners screened as negative.

**Table 1:** Distribution of risk of functional decline among SHERPA categories

	1999 cohort (≥75 years)		2009 cohort (≥75 years)	
SHERPA Category	*N*	*n* (%) decliners	*N*	*n* (%) decliners
1 = low risk	98	10 (10.2)	95	21 (22.1)
2	96	20 (20.8)	89	27 (30.3)
3	87	34 (39.1)	67	25 (37.3)
4 = high risk	74	41 (55.4)	54	29 (53.7)
	355	105 (29.6) c-statistic = 0.72 (*p* < 0.0001)	305	102 (33.4) c-statistic = 0.63 (*p* < 0.0001)

**Table 2:** SHERPA predictive performance in 2009 cohort (4 categories)

SHERPA category cut-off	positive test, %	Se, %	Sp, %	PPV, %	NPV, %	+ LR	-LR
>1	69	79	37	78	39	1.25	0.57
>2	40	53	67	74	45	1.61	0.70
>3	18	28	88	71	54	2.3	0.82

*Se* Sensitivity, *Sp* Specificity, *PPV* Positive Predictive value, *NPV* Negative Predictive value, *+LR* positive likelihood ratio, *−LR* negative likelihood ratio

In the 2009 cohort, only two of the five constitutive variables of the SHERPA score i.e., iADL and falls within a year, were significant in logistic regression analysis for functional decline, while age, self-rated health and 21-point MMSE were non-significant (Table 3). However, 21-point MMSE was kept in the model for clinical relevance (Table 4).

**Table 3:** Full prediction model, including the five constitutive variables of SHERPA

Variable	Parameter estimate	Standard error	Wald Chi²	*p*-value
Intercept	- 1.47	2.46	0.36	0.55
21-point MMSE	0.21	0.19	1.26	0.26
Fall within a year	0.51	0.27	3.77	0.05
Age	0.01	0.03	0.22	0.64
iADL	- 0.16	0.07	4.69	0.03
SRH	0.16	0.20	.062	0.43

−2 Log Likelihood intercept only = 388.734; −2 log likelihood for intercept and covariates = 369.638 (Chi² = 19.0958 [degrees of freedom = 5]; *p*-value = 0.002)

Hosmer and Lemeshow statistic (Chi² = 6.5930 [degrees of freedom = 8]; *p*-value = 0.5811)

*MMSE* Mini Mental State Evaluation, *iADL* instrumental Activity of Daily Living, *SRH* Self-rated Health

**Table 4:** Full prediction model, including iADL, falls and 21-point MMSE

Variable	Parameter estimate	Standard error	Wald Chi²	*p*-value
Intercept	−1.2974	0.2204	34.6441	< 0.0001
Fall within a year	0.5292	0.2619	4.0823	0.0433
21-point MMSE	0.2364	0.1865	1.6071	0.2049
iADL	0.3727	0.1615	5.3240	0.0210

−2 Log Likelihood intercept only = 388.734; −2 log likelihood for intercept and covariates = 371.179 (Chi² = 17.5548 [degree of freedom = 3]; *p*-value <0.001)

Hosmer and Lemeshow statistic (Chi² = 0.81 [degrees of freedom=4; *p*-value = 0.94)

*MMSE*, Mini Mental State Evaluation *iADL* instrumental Activity of Daily Living

Among other variables tested in the 2009 cohort (Table S1), only CRP - either as continuous or categorical variable (according to quartiles) - showed a statistically significant but surprisingly negative association with FD, after adjustment for SHERPA. CRP was confounded with discharge diagnosis, as defined by ICD9-CM chapters. Mean CRP was indeed 2.63 mg/dL (Standard deviation (SD) =5.24, *n* = 95) for chapter 7 (circulatory system), 6.53 mg/dL (SD = 9.63, *n* = 44) for chapter 9 (digestive system) and 2.97 mg/dL (SD = 6.25, *n* = 70) for chapter 17 (“injury and poisoning”, including falls). CRP was no longer statistically significant when these 3 discharge diagnoses were included in the model as dummy variables. CRP was therefore discarded. Finally, only one variable (discharge diagnosis ICD-9-CM, chapter 17, i.e. injury and poisoning) improved model fit (BIC = 365.65) and discrimination (c-statistic = 0.67; NRI = 0.15, z = 2.32, *p* = 0.020; IDI = 0.040, *t* = 3.27, *p* = 0.001).

## Discussion

### Main findings

In this cohort of patients aged ≥75 years, admitted through the emergency department, 102 out of 305 (33%) suffered from functional decline (FD) 3 months after discharge.

Ten years after its development, the SHERPA ability to predict 3-month FD decreased, even after re-calibration. Similarly, same discrimination ability was observed for HARP, another tool for FD prediction [17].

We did not find any additional variable, available for the 2009 cohort, i.e. demographic, comorbidity and laboratory data easily available on admission, able to improve the discrimination ability of SHERPA.

### Potential explanations for our findings

A plausible interpretation for the lower predictive yield of SHERPA in FD prediction is the change over time in some key patient characteristics in the 2009 cohort in comparison with the 1999 cohort as shown by De Brauwer et al. [25]. Both the changed case mix, which includes an increase in the frequency of geriatric features, and some differences in the process of care [25] could explain that the risk of FD in 2009 cohort patients was more uniformly distributed, and that SHERPA discriminative yield was lower than in the historical cohort (1999). These findings are coherent with other studies [10–12]. We found non-significant difference in FD rates between the 1999 and the 2009 cohorts [25].

Additional demographic and bio-clinical data available upon admission did not improve the predictive ability of the SHERPA tool. Gender and marital status were seldom cited in literature as predictors of FD [26, 27], but were not associated, in our study, with better SHERPA predictive accuracy. It has been suggested that severity of illness and geriatric syndromes, e.g. falls and their deleterious traumatic consequences, are better predictors of FD than comorbidity [26, 27]. Although the role of biological parameters as predictor of adverse functional outcomes has been studied in community-dwelling older people, evidence concerning hospitalized older patients is sparse and conflicting [6, 26, 28, 29]. de Saint Hubert showed that inflammatory bio-markers such as IL-6 and IGF-1 improved the accuracy of SHERPA by a better patient stratification [26]. However, biological parameters should be readily and quickly available on admission in order to be useful in clinical practice. In her study, CRP and albumin, also cheaper than the IL-6 and IGF-1, did not show a significant association with functional decline at 3-month, a finding consistent with our results.

Our study was limited to a single centre. Our sampling methods did not seem to produce selection bias, and consecutive eligible patients were invited to participate. Participation rate was good (9% refusal, 10% not met for practical reasons). The same medical researcher asked for informed consent, conducted the interview and phoned at 3-month the patient or his proxy. This procedure could introduce some interpretation bias of self- or proxy-reports, but can be an advantage for follow-up [30]. The geriatric evaluation and management unit (GEMU) in our institution was set up in 2002. A proportion of the frailest patients was thus admitted in this GEMU in 2009, whereas this population had to be admitted in other medical acute care units in 1999. In the present study, we excluded the patients admitted to the GEMU, as this specific geriatric setting prevents functional decline [4], the main outcome of our study.

The predictors of FD, i.e. falls, iADL and cognition, are consistent with previous studies [6–8, 27]. Whatever the method used, we were not able to add any new informative factor to the SHERPA predictive tool.

Operational definition of FD could be controversial [31]. The KATZ index definition, including incontinence, is however the most largely used instrument in the geriatric medical literature and also in clinical practice in Belgium. Appropriate time frame for measuring functional decline after hospital discharge of older people is not yet well established. As recommended in literature [31], we used pre-admission ADL score as baseline, in order to exclude FD process linked to the disease leading to hospitalization. The endpoint, namely FD, was defined at 3-month, as in the original study [13]. Definition of FD as the loss of one point in ADL score is the most used one in literature [31]. The high reliability of KATZ index was already shown, and was confirmed in our study to be very good between the two independent raters, the researcher of the 1999 cohort being the instructor of the researcher of the 2009 cohort.

## Conclusions

Predicting outcomes, as FD after several months, in older patients with a score including predictive factors easily available upon admission is a real challenge, partly because of the interaction of several independent factors, e.g. complications during the course of the hospital stay or length of stay. Acutely admitted in-patients are becoming older and their medical and functional profile is also evolving towards complexity. Complexity is the bread and butter of the geriatric field, which uses a not less complex process, namely the Comprehensive Geriatric Assessment (CGA), to deal with complexity and improve patient’s outcomes [32]. The selection of patients who will benefit from these time-consuming interventions is crucial to be efficient in a highly specialised and chronically overloaded medical ward. SHERPA and similar currently available screening tools do not offer sufficient discrimination ability, but some predictive factors stay consistent across the different tools and are related to common geriatric preoccupations, especially in ED: falls, pre-existing functional limitation and cognitive impairment. Rather than trying to summarize the patient’s frailty profile in a single score -which can probably not be improved, given the complexity of these patients- we should consider these predictors -as red flags- when evaluating older patients in the ED. Further research projects will have to take into account the limitations of the screening tools and test the efficacy of a two-step approach adapted to this setting, i.e. identification of the patients most likely to benefit from geriatric care, followed by tailored intervention.

## Additional file

Additional file 1: Table S1. Demographic, social, functional and medical data in the 305 older patients. Description of data: Demographic, social, functional and medical data in the 305 older patients. (DOC 47 kb)
