# Supplementary material for: Can we predict functional decline in hospitalized older people admitted through the emergency department? Reanalysis of a predictive tool ten years after its conception
Source: BMC Geriatr. 2017 May 12;17:105. doi: 10.1186/s12877-017-0498-0 (PMC5429553; doi:10.1186/s12877-017-0498-0)
Supplement: Additional file 1: Table S1. — Demographic, social, functional and medical data in the 305 older patients. Description of data: Demographic, social, functional and medical data in the 305 older patients. (DOC 47 kb) [file 12877_2017_498_MOESM1_ESM.doc]

| **Table 1. Demographic, social, functional and medical data in the 305 older patients** | |
| --- | --- |
| **Variables** | **Mean [DS] or n (%)** |
| Age, years*   - 75-79 - 80-84 - 85+ | 82.5 [4.9]  98 (32)  102 (33)  105 (34) |
| Sex, male* | 137 (45) |
| Residence*   - Community-dwelling - Nursing home | 283 (93)  22 (7) |
| Tertiary education* | 126 (42) |
| Marital status*   - Married - Single/separated/divorced - Widowed | 140 (46)  38 (12)  127 (42) |
| Known dementia*  MMSE (21-point) ^1^*  Self-rated Health, bad* | 25 (8)  18 [4]  25 (8) |
| bADL, KATZ ^2^*   - 6 - 4-5 - 1-3 | 5 [1]  118 (38.8)  148 (48.5)  39 (12.8) |
| iADL, LAWTON ^3^*   - 6-7 - 5 - 3-4 - 0-2 | 6 [2]  209 (68.5)  32 (10.5)  32 (10.5)  32 (10.5) |
| Medications, daily | 6 [3] |
| Comorbidity*   - CIRS^4^-total - CIRS^4^-severity - CIRS^4^-index | 15 [5]  13 [5]  5 [2] |
| Fall(s) within 1 year* | 179 (59) |
| Nutrition*   - Appetite, poor - Weight loss within 3-month - Pressure sore, yes | 64 (21)  116 (38)  5 (2) |
| Audition, poor* | 257 (84) |
| Eyesight, poor* | 260 (85) |
| Swallowing, bad* | 60 (20) |
| CRP (mg/dL) ^4^* | 5.3 [8.9] |
| GFR(mL/min) ^5^* | 59 [21] |
| Haemoglobin (g/dL)* | 12.8 [2.2] |
| Albumin (g/dL)* | 3.5 [0.5] |
| Length of stay, days* | 9 [6] |
| ICU admission ^6^* | 12 (4) |
| Discharge Residency   - Same - Rehabilitation - New Nursing Home | 217 (71)  74 (24)  8 (3) |

* Tested as predictor for functional decline; ^1^ Short Mini-Mental State: orientation (/10), attention (/5), recall (/3), learning (/3); ^2^ Basic Activity of Daily Living: bathing, dressing, walking, toileting, continence, eating (6/6=independent); ^3^ Instrumental Activity of Daily Living: telephoning, shopping, preparing meals, doing housework, using transportation, managing finances, taking medication (7/7=independent); ^4^ Cumulative Illness Rating Scale for Geriatrics; ^5^ C-Reactive Protein; ^6^ Glomerular Filtration Rate according to modification of diet in renal disease; ^7^ Intensive Care Unit
